# The impact of the Baby-Friendly Hospital Initiative on breastfeeding rates at maternity units in France

**DOI:** 10.1093/ije/dyae080

**Published:** 2024-06-10

**Authors:** Andrea Guajardo-Villar, Camille Pelat, Beatrice Blondel, Elodie Lebreton, Virginie Demiguel, Benoit Salanave, Ayoub Mitha, Hugo Pilkington, Nolwenn Regnault, ENP2021 Study Group, Camille Le Ray, Nathalie Lelong, Hélène Cinelli, Béatrice Blondel, Nolwenn Regnault, Virginie Demiguel, Elodie Lebreton, Benoit Salanave, Jeanne Fresson, Annick Vilain, Thomas Deroyon, Philippe Raynaud, Sylvie Rey, Khadoudja Chemlal, Nathalie Rabier-Thoreau, Frédérique Collombet-Migeon

**Affiliations:** Data Support, Processing and Analysis Department (DATA), French National Public Health Agency, Saint‐Maurice, France; Data Support, Processing and Analysis Department (DATA), French National Public Health Agency, Saint‐Maurice, France; Obstetric, Perinatal and Pediatric Epidemiology Research Team, Center of Research in Epidemiology and Statistics (CRESS), INSERM, Université Paris Cité, Paris, France; Non-Communicable Diseases and Trauma Department (DMNTT), French National Public Health Agency, Saint-Maurice, France; Non-Communicable Diseases and Trauma Department (DMNTT), French National Public Health Agency, Saint-Maurice, France; Non-Communicable Diseases and Trauma Department (DMNTT), French National Public Health Agency, Saint-Maurice, France; Obstetric, Perinatal and Pediatric Epidemiology Research Team, Center of Research in Epidemiology and Statistics (CRESS), INSERM, Université Paris Cité, Paris, France; Pediatric and Neonatal Intensive Care Transport Unit, Department of Emergency Medicine, SAMU 59, CHU Lille, Lille, France; Division of Clinical Epidemiology, Department of Medicine Solna, Karolinska Institutet, Stockholm, Sweden; Département de Géographie, UMR7533 Ladyss, Université Paris, Saint‐Denis, France; Non-Communicable Diseases and Trauma Department (DMNTT), French National Public Health Agency, Saint-Maurice, France

**Keywords:** Breastfeeding, Baby-Friendly Hospital Initiative, inequalities, France

## Abstract

**Background:**

The Baby-Friendly Hospital Initiative (BFHI) is associated with improved breastfeeding outcomes in many high-income countries including the UK and the USA, but its effectiveness has never been evaluated in France. We investigated the impact of the BFHI on breastfeeding rates in French maternity units in 2010, 2016 and 2021 to assess if the BFHI aids to reduce inequalities in breastfeeding.

**Methods:**

We examined breastfeeding in maternity units (exclusive, mixed and any breastfeeding) in mothers of singleton full-term newborns using the 2010 (*n* = 13 075), 2016 (*n* = 10 919) and 2021 (*n* = 10 209) French National Perinatal Surveys. We used mixed-effect hierarchical multinomial regression models adjusting for neonatal, maternal, maternity unit and French administrative department characteristics, and tested certain interactions.

**Results:**

The adjusted rate of exclusive breastfeeding was higher by +5.8 (3.4–8.1) points among mothers delivering in BFHI-accredited maternity units compared with those delivering in non-accredited units. When compared with average-weight newborns, this difference was sharper for infants with low birthweight: +14.9 (10.0–19.9) points when their birthweight was 2500 g. Mixed breastfeeding was lower by -1.7 points (-3.2–0) in BFHI-accredited hospitals, with no notable difference according to the neonatal or maternal characteristics.

**Conclusion:**

Mothers delivering in BFHI-accredited maternity units had higher exclusive breastfeeding rates and lower mixed breastfeeding rates than those delivering in non-accredited maternity units. The positive impact of the BFHI was stronger among low-birthweight neonates, who are less often breastfed, helping reduce the gap for this vulnerable group while favouring mothers with higher education levels.

Key Messages

- France has one of the lowest breastfeeding rates in Europe, with notable spatial and socioeconomic inequalities.
- The Baby-Friendly Hospital Initiative (BFHI) was first implemented in 2000 in France but there are limited data regarding its impact on breastfeeding rates.
- Overall, we found that mothers who delivered in BFHI-accredited maternity units have higher exclusive and lower mixed breastfeeding rates than mothers who delivered in non-accredited maternity units.
- The BFHI helped reduce the gap in exclusive breastfeeding rates among mothers with low-birthweight newborns.

## Introduction

Breastfeeding is pivotal for newborns’ optimal development and maternal health.^1^ Launched in 1991 by the World Health Organization (WHO) and the United Nations Children’s Fund (UNICEF), the Baby-Friendly Hospital Initiative (BFHI) promotes successful breastfeeding through 10 recommended steps.^2^ Reviews encompassing diverse settings like Israel, Taiwan, the UK and the USA, indicate increased initiation rates post-BFHI implementation.^3–5^ However, these varied contexts and study designs question generalizability.^3^ Maternal choice to breastfeed is influenced by sociodemographic and clinical factors, maternity unit practices and the socioeconomic-cultural environment.^6^ Inadequate staff training and violations of the International Code of Marketing of Breastmilk Substitutes at health care level can hinder breastfeeding, whereas adherence to the Ten Steps is crucial to prepare and support lactation.^7^ Lower socioeconomic status correlates with lower breastfeeding initiation in high-income countries.^8^^,^.^9^ Mode of delivery, birthweight and gestational age are predictors of breastfeeding initiation in countries like France, Spain, and Brazil.^10–12^ In addition to the general guidelines, the BFHI also provides a guide to support breastfeeding among ‘small, sick and preterm babies’,^13^ but no specific recommendation based on the social, demographic or clinical characteristics of mothers.

Breastfeeding rates in French maternity units, among Europe’s lowest, decreased over 2010–16, plateauing in 2021.^14–16^ With nearly all French births in maternity units,^17^ interventions targeting maternity units can significantly affect breastfeeding rates because of a privileged action window.^18^ In France, those maternity units that prove their compliance with the national version of the ‘Baby-Friendly’ guidelines, comprising 12 recommendations (see Supplementary Table S1, available as Supplementary data at *IJE* online), and agree to a yearly evaluation, earn a 4-year accreditation.^19^ The BFHI in France focuses on supporting newborns and their families and does not require a minimum rate of exclusive breastfeeding.^19^ France obtained its first BFHI-accredited maternity unit in 2000. In 2021, 49 out of 456 maternity units (11%) were accredited,^19^ but data on the impact of the initiative remain scarce. In a study of breastfeeding trends in France between 2010 and 2016, we reported a positive association between the BFHI and breastfeeding rates in maternity units, as exclusive breastfeeding rates were 8.1 points higher in accredited maternity units [95% confidence interval (CI): 4.6, 11.3).^16^

This study analyses the BFHI’s association with breastfeeding rates in French maternity units, using the 2010, 2016 and 2021 French National Perinatal Surveys (Enquête Nationale Périnatale, ENP), considering individual and contextual confounding factors. The study also aims to assess the BFHI's role in reducing breastfeeding inequalities among different mother-infant subgroups with varying socioeconomic, demographic and clinical characteristics.

## Methods

### Study population

Data on singleton full-term newborns, their mothers and maternity units in metropolitan France from the 2010, 2016 and 2021 ENP were used; their study design is described elsewhere.^20^Supplementary Figure S1 (available as Supplementary data at *IJE* online) presents the exclusion criteria in our study. Our final study population included 522 maternity units and 13 075 mother–infant pairs in 2010, 493 maternity units and 10 919 mother–infant pairs in 2016 and 456 maternity units and 10 209 mother–infant pairs in 2021.

### Definitions and variables

Breastfeeding was self-declared [‘How is your child fed today?’ 1, only breastmilk,; or 2, only formula; or 3, mixed breastfeeding (breastmilk and formula) in all survey editions] in the interview that took place during the first days of the maternity stay, see details in Supplementary Table S2 (available as Supplementary data at *IJE* online). Based on this question, we created the breastfeeding variable with three categories: exclusive (only breast milk); mixed (breast milk and newborn or preterm formula); and no breastfeeding (only newborn or preterm formula). ‘Any breastfeeding’, used from here on, refers to the addition of exclusive and mixed breastfeeding.

The explanatory variable of main interest was the BFHI accreditation status at the time the ENP took place. The maternity units that were in the process of obtaining their accreditation were classified as non-accredited (20 in 2010, 40 in 2016 and 31 in 2021). Supplementary Table S3 (available as Supplementary data at *IJE* online) shows the number of maternity units by BFHI-accreditation status per year and in total. The other variables were the survey year and variables previously identified to be associated with breastfeeding in France.^16^ Individual variables comprised maternal age, maternal level of education, maternal country of birth, marital status, average monthly household income, employment during pregnancy, parity (i.e. number of births before this delivery), pre-pregnancy body mass index (BMI in kg/m^2^), mode of delivery, the time between giving birth and the survey interview (in days), birthweight (g), gestational age (weeks) and neonatal transfer. Maternity unit variables were the size (annual number of deliveries), status and level of care. The characteristics of the French administrative department where the maternity unit was located comprised the percentage of immigrants, the percentage of residents aged 16 years and over with a graduate or post-graduate education, and the percentage of urban population. Details on modalities, sources and variable calculations are given in Supplementary Methods (available as Supplementary data at *IJE* online).

### Statistical analysis

The proportion of mothers with at least one missing value for one of the variables of interest was 13.5% in 2010, 6.0% in 2016 and 13.2% in 2021. To avoid estimation biases potentially induced by the complete-case design, we imputed missing values using the missForest method, which builds random forest models to impute missing values in continuous and categorical variables.^21^

We then modelled breastfeeding rates (exclusive, mixed, no) through five multinomial regression models, using exclusive breastfeeding as the reference.^22^ Model one only included the BFHI accreditation and the year of the survey. Then, we progressively added the individual (Model two), maternity unit (Model three) and French administrative department (Model four) characteristics. Continuous variables (maternal age, pre-pregnancy BMI, time between giving birth and the survey interview, birthweight, gestational age, maternity unit size and the French administrative department characteristics) were modelled with smoothing splines to account for non-linear effects.^23^ Model four also included a spatially-structured random effect at the French administrative department level to account for the unexplained variations of breastfeeding rates between departments, as well as the correlation between breastfeeding rates of neighbouring departments.^24^

For each model, we used backward elimination to remove the covariates with a *P*>=0.05 and the smooth terms whose confidence interval included zero when plotted.^25^^,^^26^ Then, based on the literature, we tested potential interactions between the BFHI accreditation status and maternal or neonatal characteristics (survey year, maternal education level, maternal country of birth, average monthly household income, parity, mode of delivery, time between giving birth and the survey interview, birthweight and gestational age) and included those with *P *< 0.1. Thus Model five, the final model, included two interactions terms (BFHI accreditation status and maternal education level; BFHI accreditation status and birthweight).

We produced exclusive, mixed and any breastfeeding marginal predictions, for each level of the covariates in the final model to ease interpretation. Marginal predictions are the mean of predicted responses calculated by replacing the values of a covariate with a specific hypothetical value (e.g. parity is fixed to one for all mothers) while all other covariates remain unchanged.^27^ We also present the marginal effects, or the difference between the predicted breastfeeding rates with each variable level and the reference level.^27^ We computed 95% CIs for the marginal predictions and effects using 1000 Monte-Carlo simulations. All analyses were performed with R statistical software v4.1.3,^28^ and the mgcv v1.8–31,^23^ and missForest v1.4 packages.^29^

## Results

### Description of newborns, mothers and maternity units

The cumulated number of interviewed mothers over the 3 years by French administrative department ranged from 24 in the French administrative department of La Creuse to 1915 in Paris. Table 1 describes the feeding practices and the sociodemographic and clinical characteristics of mothers and newborns, as well as the characteristics of the maternity units by BFHI accreditation status and survey time. In bivariate analyses, mothers giving birth in BFHI-accredited maternity units were most frequently born in France, unmarried and their newborn had less frequent neonatal transfer in 2016 and 2021, see Table 1 Crude exclusive breastfeeding rates were higher in accredited than in non-accredited maternity units, and mixed breastfeeding rates were lower.

**Table 1.** Distribution of the characteristics of mothers and singleton newborns in the French National Perinatal Surveys [Enquête Nationale Périnatale (ENP)], by year and maternity unit Baby-Friendly Hospital Initiative accreditation status

	Survey year								
	2010			2016			2021		
Characteristic	Non-accredited (*n *=* *12 818)	Accredited (*n *=* *257)	***P*** *^a^*	Non-accredited (*n *=* *10 217)	Accredited (*n *=* *702)	***P*** *^a^*	Non-accredited (*n *=* *9193)	Accredited (*n *=* *1016)	***P*** *^a^*
Breastfeeding			0.09			<0.01			<0.01
Exclusive	7976 (62.2%)	177 (68.9%)		5525 (54.1%)	436 (62.1%)		5108 (55.6%)	622 (61.2%)	
Mixed	946 (7.38%)	15 (5.84%)		1347 (13.2%)	35 (4.99%)		1349 (14.7%)	70 (6.89%)	
None	3896 (30.4%)	65 (25.3%)		3345 (32.7%)	231 (32.9%)		2736 (29.8%)	324 (31.9%)	
Maternal age (years)			0.52			0.59			0.94
<25	2108 (16.4%)	35 (13.6%)		1297 (12.7%)	96 (13.7%)		1009 (11.0%)	113 (11.1%)	
25–29	4291 (33.5%)	86 (33.5%)		3272 (32.0%)	213 (30.3%)		2602 (28.3%)	292 (28.7%)	
30–34	3980 (31.1%)	80 (31.1%)		3496 (34.2%)	252 (35.9%)		3337 (36.3%)	372 (36.6%)	
≥35	2439 (19.0%)	56 (21.8%)		2152 (21.1%)	141 (20.1%)		2245 (24.4%)	239 (23.5%)	
Level of education			0.05			0.34			0.42
No/primary	296 (2.31%)	4 (1.56%)		159 (1.56%)	12 (1.71%)		158 (1.72%)	11 (1.08%)	
Lower secondary	3248 (25.3%)	78 (30.4%)		2150 (21.0%)	167 (23.8%)		1561 (17.0%)	158 (15.6%)	
Upper secondary	2541 (19.8%)	38 (14.8%)		2215 (21.7%)	136 (19.4%)		1981 (21.5%)	228 (22.4%)	
1–2 years university	2770 (21.6%)	46 (17.9%)		1961 (19.2%)	127 (18.1%)		1697 (18.5%)	192 (18.9%)	
>2 years university	3963 (30.9%)	91 (35.4%)		3732 (36.5%)	260 (37.0%)		3796 (41.3%)	427 (42.0%)	
Country of birth			0.08			<0.01			<0.01
France	10 465 (81.6%)	221 (86.0%)		8299 (81.2%)	616 (87.7%)		7245 (78.8%)	875 (86.1%)	
African country	1468 (11.5%)	18 (7.00%)		1214 (11.9%)	41 (5.84%)		679 (7.39%)	46 (4.53%)	
Other country	885 (6.90%)	18 (7.00%)		704 (6.89%)	45 (6.41%)		1269 (13.8%)	95 (9.35%)	
Marital status^b^			0.54			<0.01			<0.01
Not married	6711 (52.4%)	140 (54.5%)		6036 (59.1%)	459 (65.4%)		5639 (61.3%)	683 (67.2%)	
Married	6107 (47.6%)	117 (45.5%)		4181 (40.9%)	243 (34.6%)		3554 (38.7%)	333 (32.8%)	
Average monthly household income (€)^c^			0.99			0.64			0.73
<1500	2673 (20.9%)	54 (21.0%)		1873 (18.3%)	137 (19.5%)		1451 (15.8%)	156 (15.4%)	
1500–2999	5712 (44.6%)	115 (44.7%)		4098 (40.1%)	284 (40.5%)		3028 (32.9%)	347 (34.2%)	
≥3000	4433 (34.6%)	88 (34.2%)		4246 (41.6%)	281 (40.0%)		4714 (51.3%)	513 (50.5%)	
Employment during pregnancy			0.83			0.50			0.78
No	3794 (29.6%)	74 (28.8%)		2956 (28.9%)	212 (30.2%)		2749 (29.9%)	299 (29.4%)	
Yes	9024 (70.4%)	183 (71.2%)		7261 (71.1%)	490 (69.8%)		6444 (70.1%)	717 (70.6%)	
Parity (number of births before this one)			0.18			0.83			0.67
Primiparous	5477 (42.7%)	107 (41.6%)		4299 (42.1%)	297 (42.3%)		3794 (41.3%)	433 (42.6%)	
1	4485 (35.0%)	101 (39.3%)		3727 (36.5%)	253 (36.0%)		3302 (35.9%)	372 (36.6%)	
2	1892 (14.8%)	27 (10.5%)		1455 (14.2%)	108 (15.4%)		1330 (14.5%)	135 (13.3%)	
3	638 (4.98%)	12 (4.67%)		459 (4.49%)	28 (3.99%)		487 (5.30%)	50 (4.92%)	
4 or more	326 (2.54%)	10 (3.89%)		277 (2.71%)	16 (2.28%)		280 (3.05%)	26 (2.56%)	
Pre-pregnancy BMI (kg/m²)			0.80			0.03			0.77
<18.5	985 (7.68%)	17 (6.61%)		723 (7.08%)	66 (9.40%)		520 (5.66%)	59 (5.81%)	
18.5–24.9	7859 (61.3%)	164 (63.8%)		5867 (57.4%)	384 (54.7%)		4874 (53.0%)	549 (54.0%)	
25–29.9	2670 (20.8%)	53 (20.6%)		2352 (23.0%)	150 (21.4%)		2374 (25.8%)	263 (25.9%)	
≥30	1304 (10.2%)	23 (8.95%)		1275 (12.5%)	102 (14.5%)		1425 (15.5%)	145 (14.3%)	
Time between delivery and interview (days)			<0.01			<0.01			0.13
0	1823 (14.2%)	19 (7.39%)		1529 (15.0%)	109 (15.5%)		957 (10.4%)	114 (11.2%)	
1	6229 (48.6%)	83 (32.3%)		5302 (51.9%)	310 (44.2%)		5138 (55.9%)	533 (52.5%)	
2	2982 (23.3%)	73 (28.4%)		2438 (23.9%)	214 (30.5%)		2433 (26.5%)	277 (27.3%)	
3	1328 (10.4%)	55 (21.4%)		740 (7.24%)	57 (8.1%)		564 (6.14%)	76 (7.48%)	
≥4	456 (3.56%)	27 (10.5%)		208 (2.04%)	12 (1.7%)		101 (1.10%)	16 (1.57%)	
Mode of delivery			0.13			0.75			0.06
Spontaneous vaginal	8761 (68.3%)	190 (73.9%)		7111 (69.6%)	495 (70.5%)		6262 (68.1%)	693 (68.2%)	
Instrumental	1637 (12.8%)	24 (9.34%)		1288 (12.6%)	90 (12.8%)		1177 (12.8%)	152 (15.0%)	
Caesarean section	2420 (18.9%)	43 (16.7%)		1818 (17.8%)	117 (16.7%)		1754 (19.1%)	171 (16.8%)	
Birthweight (g)			0.63			0.13			0.07
<2500	278 (2.17%)	7 (2.72%)		266 (2.60%)	15 (2.14%)		215 (2.34%)	16 (1.57%)	
2500–2999	2336 (18.2%)	53 (20.6%)		1937 (19.0%)	151 (21.5%)		1646 (17.9%)	184 (18.1%)	
3000–3499	5574 (43.5%)	108 (42.0%)		4359 (42.7%)	296 (42.2%)		3970 (43.2%)	414 (40.7%)	
3500–3999	3653 (28.5%)	66 (25.7%)		2861 (28.0%)	201 (28.6%)		2682 (29.2%)	307 (30.2%)	
≥4000	977 (7.62%)	23 (8.95%)		794 (7.77%)	39 (5.56%)		680 (7.40%)	95 (9.35%)	
Gestational age (weeks)			0.50			0.17			0.02
37	825 (6.44%)	23 (8.95%)		698 (6.83%)	62 (8.83%)		570 (6.20%)	51 (5.02%)	
38	2221 (17.3%)	37 (14.4%)		1709 (16.7%)	101 (14.4%)		1616 (17.6%)	157 (15.5%)	
39	3420 (26.7%)	68 (26.5%)		2985 (29.2%)	217 (30.9%)		2760 (30.0%)	293 (28.8%)	
40	3782 (29.5%)	81 (31.5%)		2891 (28.3%)	184 (26.2%)		2469 (26.9%)	292 (28.7%)	
41	2523 (19.7%)	47 (18.3%)		1881 (18.4%)	134 (19.1%)		1724 (18.8%)	221 (21.8%)	
42	47 (0.37%)	1 (0.39%)		53 (0.52%)	4 (0.57%)		54 (0.59%)	2 (0.20%)	
Neonatal transfer			0.35			0.01			<0.01
No	12237 (95.5%)	249 (96.9%)		9700 (94.9%)	682 (97.2%)		8705 (94.7%)	984 (96.9%)	
Yes	581 (4.53%)	8 (3.11%)		517 (5.06%)	20 (2.85%)		488 (5.31%)	32 (3.15%)	

BMI, body mass index.

a The presented *P-*values were obtained with chi square tests.

b The binary variable of marital status was based on the question ‘Are you married?’ and had two possible answers in 2010 (no or yes) and three in 2016 and 2021 (no, yes, in civil union). We grouped ‘civil union’ and ‘no' in 2016 and 2021, to be consistent with the 2010 French National Perinatal Survey report.

c The average monthly household income (€) in 2021 was after income tax, in 2010 and 2016 before income tax.

**Figure 1.**
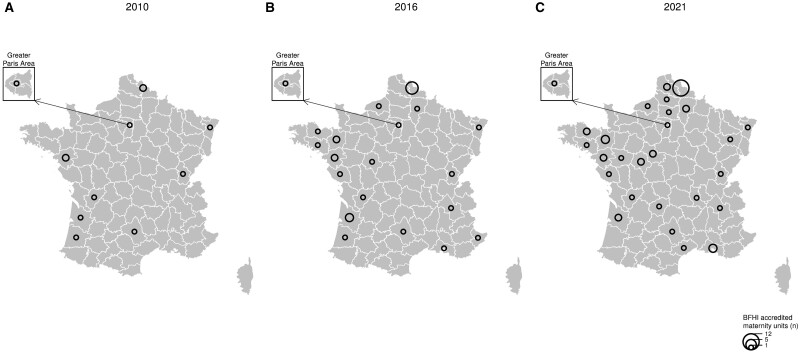
Number of Baby-Friendly Hospital Initiative (BFHI)-accredited maternity units by French administrative departments in 2010 (A), 2016 (B) and 2021 (C) in metropolitan France. Data source: BFHI France

There were 11 accredited maternity units in 2010 (2%), 29 in 2016 (6%) and 48 in 2021 (11%). Figure 1 shows on a map the number of BFHI-accredited maternity units by French administrative department by survey year.

Supplementary Table S3 (available as Supplementary data at *IJE* online) outlines the number of maternity units included, their BFHI accreditation status and their characteristics. Most of the BFHI-accredited maternity units have a status of ‘other public’ (not public regional or university), level of care I (with an obstetrics ward) and a size of less than 2000 annual deliveries.

### Impact of the BFHI on breastfeeding rates

In our final model, the BFHI was associated with increased rates of exclusive breastfeeding [+5.8 percentage points (95% CI, 3.4, 8.1)] and any breastfeeding [+4.1 (95% CI, 2.0, 6.2)] and with decreased rates of mixed breastfeeding [-1.7 (95% CI, -3.2, 0)].

**Figure 2.**
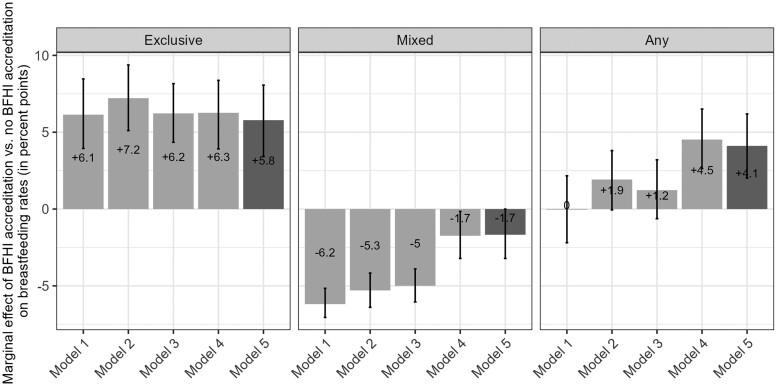
Difference in predicted exclusive (only breastmilk), mixed (breastmilk and formula) and any (addition of exclusive and mixed) breastfeeding rates in maternity units between mothers who delivered in a Baby-Friendly Hospital Initiative (BFHI)-accredited maternity unit and those that delivered in non-BFHI-accredited maternity units, or the marginal effect of the BFHI. Any breastfeeding rates are equal to the sum of exclusive and mixed breastfeeding rates. The five nested models cumulatively adjusted for the BFHI and the year (model one), the individual characteristics (Model two), the maternity unit characteristics (Model three), the French administrative department characteristics and random effect (Model four) and interaction terms between the BFHI and some individual characteristics (Model five). Data source: 2010, 2016 and 2021 French Perinatal National Surveys

To investigate the impact of adjusting on covariates on these estimates, we present in Figure 2 the marginal effect of the BFHI on breastfeeding rates for all five models. We also present the marginal predictions per model for BFHI-accredited maternity units in Supplementary Table S4 (available as Supplementary data at *IJE* online) and for non-accredited in Supplementary Table S5 (available as Supplementary data at *IJE* online). Supplementary Table S6 (available as Supplementary data at *IJE* online) outlines the variables included and selected for each modelling step. Once selected, the variables remained significant in all subsequent modelling steps.

Model one included only the survey year and the BFHI status. It predicted that mothers would exclusively breastfeed +6.1 (95% CI, 3.9, 8.5) percentage points if they had given birth in a BFHI-accredited maternity unit than if they had delivered in a non-accredited maternity unit. This estimate remained quite stable throughout all other models.

Regarding mixed breastfeeding, the marginal effect of the BFHI inMmodel one was -6.2 (95% CI, -7.1, -5.2) points and increased to -1.7 (95% CI, -3.2, 0) points in Model five. We observed the most important change when adjusting for the characteristics and the random effect of the French administrative departments: the marginal effect of the BFHI then flattened towards zero.

The BFHI was associated with an increase in the rate of any breastfeeding that ranged from 0 points (95% CI, -2.2, 2.2) in the unadjusted model (Model 1) to +4.1 points (95% CI, 2.0, 6.2) in the fully adjusted model (Model 5). Noticeably, the 95% CI of this marginal effect excluded the zero once the model included the spatial random effect (Model 4).

### Impact of other covariates

**Figure 3.**
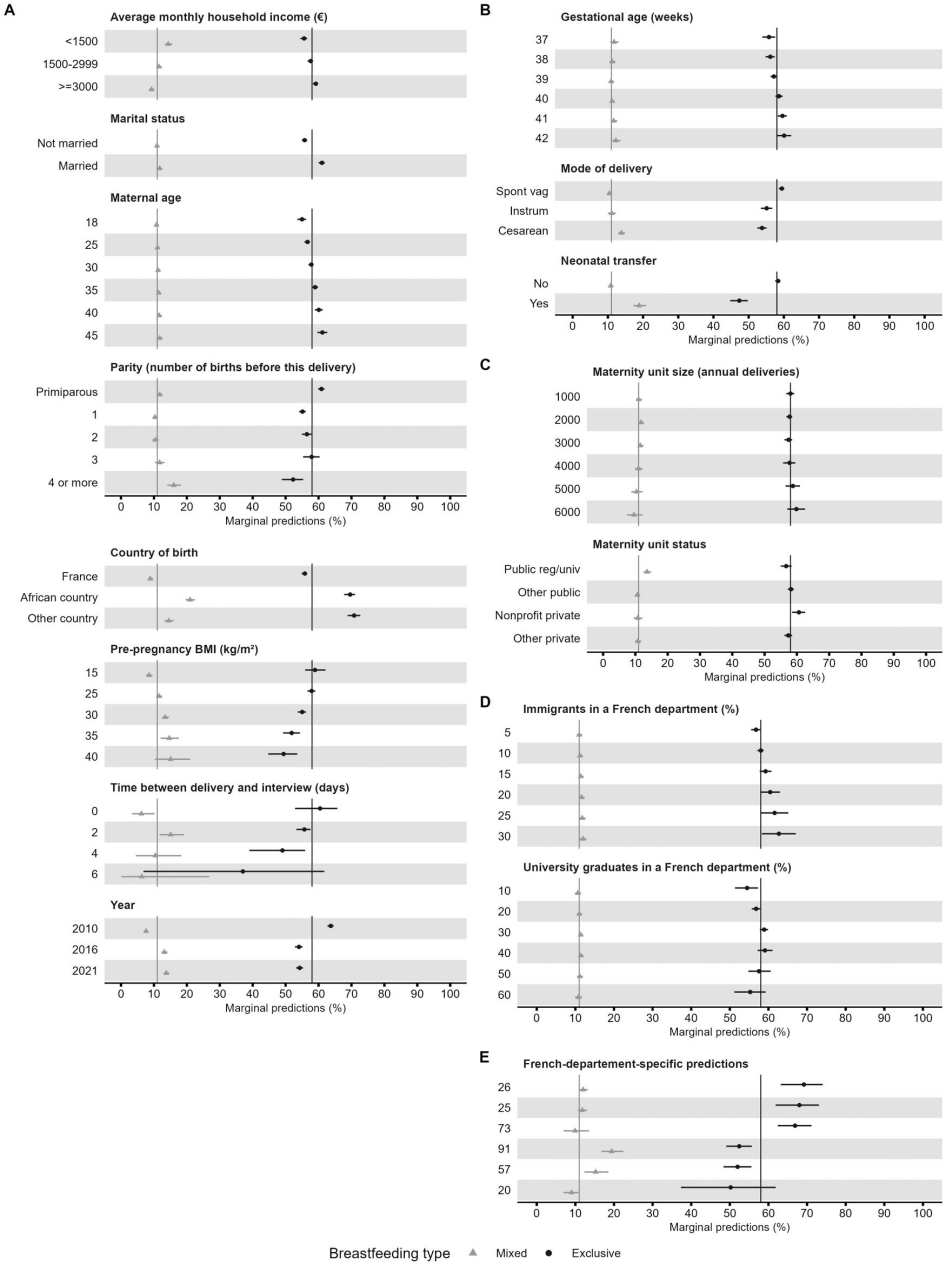
Marginal predictions of mixed breastmilk and formula (triangle) and exclusive, only breastmilk (circle) breastfeeding rates in maternity units for the variables included in the final model (Model five). The final model included the Baby-Friendly Hospital Initiative (BFHI), neonatal, maternal, maternity unit and French administrative department characteristics, as well as a spatial random effect at the department level, and interactions terms between the BFHI and the maternal education level and neonatal birthweight. Section (A) shows the predictions against the maternal variables, (B) the newborn variables, (C) the maternity unit variables, (D) the French administrative department variables and (E) the French administrative department-specific random effect for the three highest and lowest values. The vertical lines indicate the mean marginal prediction for each type of breastfeeding. Data source: 2010, 2016 and 2021 French Perinatal National Surveys

Figure 3 presents the marginal predictions of exclusive and mixed breastfeeding rates based on the final Model five for different values of the adjusting covariates. The marginal effects are presented in Supplementary Table S7 (available as Supplementary data at *IJE* online). Maternal and neonatal characteristics had stronger associations with breastfeeding rates than maternity unit characteristics. The variation in breastfeeding rates according to French administrative department characteristics was visible but smaller than the unexplained variation between departments, computed from the random effect, see Figure 3E; and Supplementary Figures S2 and S3 (available as Supplementary data at *IJE* online).

### Impact of the BFHI by subgroups of mothers

**Figure 4.**
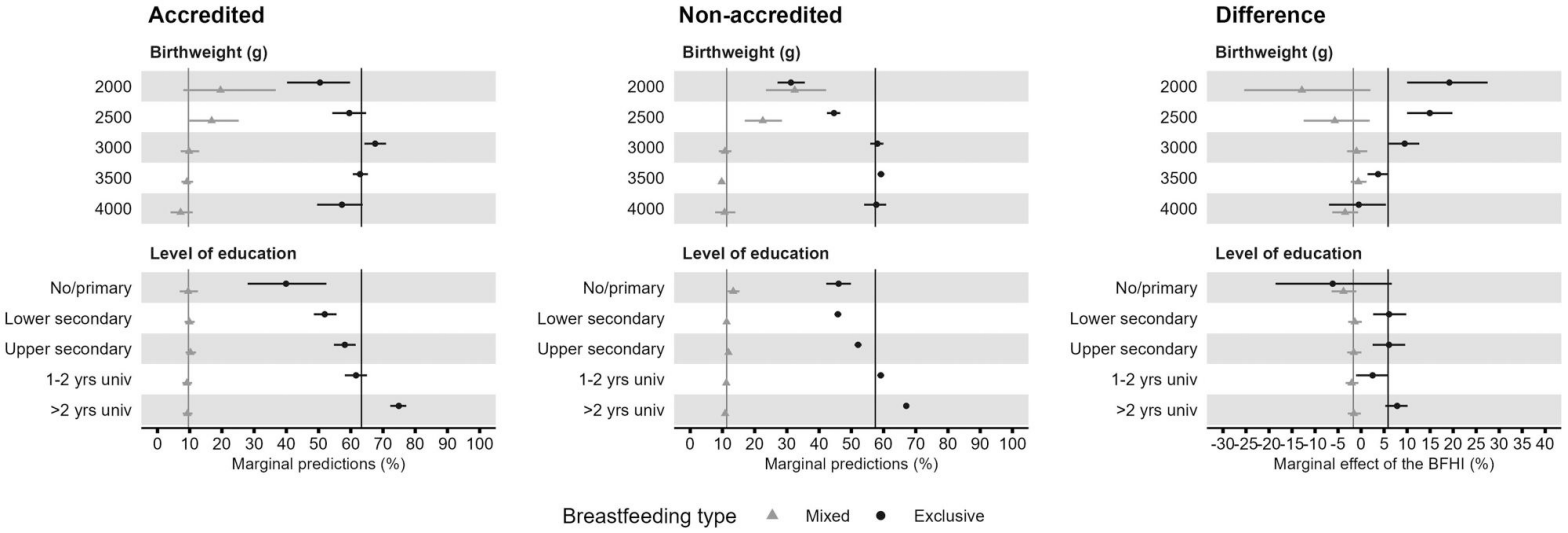
Marginal predictions of mixed breastmilk and formula (triangle) and exclusive, only breastmilk (circle) breastfeeding rates in maternity units for the variables in interaction with the Baby-Friendly Hospital Initiative (BFHI) in the final model (Model 5). The ‘Accredited’ and ‘Non-accredited’ sections present the marginal predictions for mothers who delivered in a BFHI-accredited and non-accredited maternity unit, respectively. The section ‘Difference’ presents the marginal effects, that is, the differences in predicted breastfeeding initiation rates between accredited and non-accredited maternity units. The vertical lines indicate the mean marginal prediction for each type of breastfeeding. The horizontal lines across the triangles or circles represent the 90% confidence interval. The model included the BFHI variable, neonatal, maternal, maternity unit and French administrative department characteristics, as well as a spatial random effect at the French administrative department level, and interactions terms between the BFHI and the maternal education level and neonatal birthweight. Data source: 2010, 2016 and 2021 French Perinatal National Surveys

Figure 4 presents the predicted breastfeeding rates in BFHI-accredited and non-accredited maternity units, and the difference between those rates (also called the ‘marginal effect’ of BFHI) for different levels of maternal education level and birthweight, two variables interacting with the BFHI in Model five. Values are shown in Supplementary Table S8 (available as Supplementary data at *IJE* online). In short, the BFHI marginal effect on exclusive breastfeeding rates increased with maternal education: from -6.2 (95% CI, -18.6, 6.7) points for mothers with no or primary education to +7.8 (95% CI, 5.2, 10.1) points for mothers with 2 or more years of university education. The BFHI marginal effect on exclusive breastfeeding rates increased as the newborn birthweight decreased: from +3.7 (95% CI, 1.4, 6.0) points for newborns weighing 3500 g to +14.9 (95% CI, 10.0, 19.9) points if the birthweight was 2500 g.

## Discussion

We showed with adjusted models that BFHI-accredited maternity units had higher rates of exclusive and any breastfeeding, and lower rates of mixed breastfeeding, in metropolitan France in 2010, 2016 and 2021. The adjustment on covariates, and above all on a spatial random effect, was necessary to unveil the strong positive association between the BFHI and any breastfeeding rates, as it reduced the decrease in mixed breastfeeding rates observed in BFHI-accredited units in the crude analysis. This can be explained by the territorial disparities of breastfeeding rates in metropolitan France and the fact that BFHI-accredited maternity units are unevenly distributed across this territory, as exemplified by the high concentration of accredited maternity units in the Nord (the northernmost French administrative department), which engaged from the early days of the BFHI in France.^19^

### Impact of the BFHI by birthweight

The positive association between the initiative and exclusive breastfeeding rates was stronger in mothers of low-birthweight newborns. In our results, in line with previous results from France, Spain and Brazil,^10^^,^^12^^,^^30^ low-birthweight newborns are less breastfed than average-weight newborns. A lack of appropriate advice on lactation, stress management and infant behaviour from health professionals during the first days after birth, along with the marketing of commercial milk formula, may lead parents to introduce formula.^7^ We found that the increase in exclusive breastfeeding and the decrease in mixed breastfeeding rates associated with the BFHI accreditation were greater among low-birthweight newborns, helping reduce the existing gap for this vulnerable group. To our knowledge, this is the first report of a different impact of the BFHI according to birthweight. These results indicate that, for these newborns, the staff practices in the accredited maternity units align with the ‘Ten steps to successful breastfeeding’ published in 2018,^18^ particularly Step 2 ‘Staff competency’ (‘Help a mother to breastfeed a low-birthweight’) and Step 6 ‘Supplementation’ (‘Do not provide breastfed newborns any food or fluids other than breast milk, unless medically indicated’). This may also be the successful result of the BFHI publishing dedicated breastfeeding support guidelines for ‘low-birthweight, preterm and sick newborns’.^13^

### Impact of the BFHI by maternal educational level

The BHFI had a positive impact on breastfeeding rates in mothers with average and high education levels. For mothers with no or primary education, the rate of exclusive breastfeeding was lower in accredited maternity units than in non-accredited ones, although with a large confidence interval. In France, these mothers are part of a minority group with specific characteristics: for example, they are more likely to be born outside France and to have lower household incomes. However, disentangling the association of each of these characteristics with breastfeeding would require a larger sample size. A cross-sectional study in Belgium also reported that the initiative improved exclusive breastfeeding initiation rates mostly in the subgroups of mothers who were already more likely to breastfeed (particularly those with higher educational levels) but did not differentiate mothers with no or primary education.^31^ Conversely the BFHI in Maine, USA, helped increase breastfeeding initiation rates by 8.6 percentage points in mothers with lower education level vs no difference noticeable in mothers with higher education level.^32^

### Strengths and limitations

To our knowledge, this is the first study to evaluate the impact of the BFHI on breastfeeding rates in maternity units at a national level in France. We used data from the 2010, 2016 and 2021 ENP, which allowed us to examine the impact of the BFHI throughout time and todetermine that there was no noticeable change of the association between BFHI and breastfeeding over time (no interaction BFHI × survey year). The ENP data are collected by trained midwives, using the same methodology in nearly all French maternity units, with few missing data, and can be considered representative of all births in France.^20^ Another strength is that we differentiated exclusive and mixed breastfeeding rates, when most studies in France only use any breastfeeding rates.^33^^,^^34^ Our results can be enriched by the analysis of the impact of the BFHI on breastfeeding continuation and duration using the data of the ENP 2021 at 2, 6 and 12 months.

We grouped the maternity units that were in the process of obtaining the BFHI-accreditation with those that were not accredited, see Supplementary Table S9 (available as Supplementary data at *IJE* online). As a sensitivity analysis, we computed adjusted breastfeeding rates categorizing the BFHI accreditation status into three categories (accredited, in process, not accredited), see Supplementary Table S10 (available as Supplementary data at *IJE* online). We observed that exclusive and any breastfeeding rates in the maternity units that were in process of obtaining the BFHI accreditation fell midway between those in accredited and non-accredited maternity units. Keeping only two categories of BFHI accreditation, a common choice in the literature, allowed us to clearly study the interactions between the BFHI accreditation status and the characteristics of newborns and mothers, one of our main objectives.

An innate limitation of this study is the small number of BFHI-accredited maternity units. Whereas our results demonstrate a positive association between delivering in a BFHI-accredited maternity unit and exclusive and any breastfeeding rates, the cross-sectional design of our study provides lower level of proof than a randomized controlled trial design would. A limitation of the survey question used to measure breastfeeding at the maternity unit, ‘How is your child fed today?’, is that it does not specify a time frame (within 1 h of birth), as the question used by the WHO to measure breastfeeding initiation does.^35^ However, the question remained unchanged through the 2010, 2016 and 2021 editions of the ENP, allowing us to measure breastfeeding in the same way. As most mothers were interviewed within 1 day after delivery, we found it reasonable to use the question as a proxy for breastfeeding initiation at the maternity unit.

Finally, international comparisons should take into account that some of the ‘Ten steps to successful breastfeeding’ have been adapted in France and that two additional steps were added,^36^ see Supplementary Table S1 (available as Supplementary data at *IJE* online).

## Conclusion

This repeated cross-sectional study showed that mothers delivering in a BFHI-accredited maternity unit had higher adjusted rates of exclusive [+5.8 percentage points (95% CI, 3.4, 8.1)) and any (+4.1 (95% CI, 2.0, 6.2)) breastfeeding, and lower mixed (-1.7 (95% CI, -3.2, 0)] breastfeeding rates than mothers in non-accredited maternity units in metropolitan France. The BFHI helped narrow the gap in exclusive breastfeeding rates of low-birthweight newborns, who are less often breastfed, while favouring mothers with higher education levels, already the most likely to breastfeed.

## The ENP 2021 Study Group

Camille Le Ray (Inserm EPOPé), Nathalie Lelong (Inserm EPOPé), Hélène Cinelli (Inserm EPOPé), Béatrice Blondel (Inserm EPOPé), Nolwenn Regnault (Santé publique France), Virginie Demiguel (Santé publique France), Elodie Lebreton (Santé publique France), Benoit Salanave (Santé publique France), Jeanne Fresson (Direction de la Recherche, des Etudes, de l’Evaluation et des Statistiques), Annick Vilain (Direction de la Recherche, des Etudes, de l’Evaluation et des Statistiques), Thomas Deroyon (Direction de la Recherche, des Etudes, de l’Evaluation et des Statistiques), Philippe Raynaud (Direction de la Recherche, des Etudes, de l’Evaluation et des Statistiques), Sylvie Rey (Direction de la Recherche, des Etudes, de l’Evaluation et des Statistiques), Khadoudja Chemlal (Direction Générale de la Santé), Nathalie Rabier-Thoreau (Direction Générale de la Santé), Frédérique Collombet-Migeon (Direction Générale de l’Offre de Soin).

## Ethics approval

The French national perinatal surveys [Enquête Nationale Périnatale (ENP)] received approval from the relevant legal authorities in France at the time each survey took place. For the 2010 and 2016 ENP these included: the ethics commitee of the Institut National de la Santé et de la Recherche Médicale (INSERM) and the Commission Nationale de l’Informatique et des Libertés (CNIL) for data protection and confidentiality, the Comité Naitonal de l’Information Statistique (CNIS) for statistical quality control, and the Comité consultative sur le Traitement de l’Information en matière de Recherche dans le domaine de la Santé (CCTIRS). For the 2021 ENP these included: the Label Committee, the Committee for the Protection of Persons (CPP), the Committee of Ethics and Scientists for Research, Studies and Evaluations (CESREES) and the Commission Nationale de l’Informatique et des Libertés (CNIL).

## Supplementary Material

dyae080_Supplementary_Data

## Data Availability

Data from the French national perinatal surveys [Enquête Nationale Périnatale (ENP)] are not shared. The list of the Baby-Friendly Hospital Initiative (BFHI) accreditation status per year was provided by the BFHI France. The data sources for the French administrative department characteristics are openly available in the French National Institute of Statistics and Economical Studies (INSEE) at [https://www.insee.fr/], with the following references: Education in 2010. Population census—detailed tables (internet). 2013 (cited 21 June 2019). Available from: [https://www.insee.fr/fr/statistiques/2053487?sommaire=2118583]. Education in 2016. Population census—detailed tables (internet). 2019. Available from: [https://www.insee.fr/fr/statistiques/4171399?sommaire=4171407]. Education in 2019. Population census—detailed tables (internet). 2019. Available from: [https://www.insee.fr/fr/statistiques/6454124?sommaire=6454135]. Legal population in France in 2010 (internet). 2012. Available from: [https://www.insee.fr/fr/statistiques/2119780?sommaire=2128804#titre-bloc-2]. Legal population in France in 2016 (I]internet). 2018. Available from: [https://www.insee.fr/fr/statistiques/3677785?sommaire=3677855]. Legal population in France in 2020 (internet). 2023. Available from: [https://www.insee.fr/fr/statistiques/6683035?sommaire=6683037]. Nationality and immigration in 2010. Population census—detailed tables (internet). 2013 (cited 24 September 2019). Available from: [https://www.insee.fr/fr/statistiques/2053205?sommaire=2403632]. Nationality and immigration in 2016. Population census—detailed tables (internet). 2019 (cited 21 June 2019). Available from: [https://www.insee.fr/fr/statistiques/4171508?sommaire=4171510]. Nationality and immigration in 2019–20. Population census—detailed tables (internet). 2020. Available from: [https://www.insee.fr/fr/statistiques/3633212?msclkid=caa0b899aa8511ecbfe3152a7ca4344d]. Urban units database in France in 2010 (internet). 2019. Available from: [https://www.insee.fr/fr/information/2115018]. Urban units database in France in 2020 (internet). 2022. Available from: [https://www.insee.fr/fr/information/4802589].
