# Supplementary material for: The impact of the Baby-Friendly Hospital Initiative on breastfeeding rates at maternity units in France
Source: Int J Epidemiol. 2024 Jun 10;53(3):dyae080. doi: 10.1093/ije/dyae080 (PMC11164413; doi:10.1093/ije/dyae080)
Supplement: dyae080_Supplementary_Data [file dyae080_supplementary_data.docx]

Supplementary Material

**Supplementary Tables…………………………………………………………….…………………......1-10**

**Supplementary Table S1**. The 12 recommendations of the Baby-Friendly Hospital Initiative (BFHI) in France, translated to English and in the original French version.……………………………………………………...……….…...…….………**1**

**Supplementary Table S2.** Survey questions of the French Perinatal National Surveys (ENP) used to measure breastfeeding at the maternity unit translated to English and in the original French version………………………………………....…..….**2**

**Supplementary Table S3.** Distribution of the characteristics of maternity units by year and their Baby-Friendly Hospital Initiative (BFHI) accreditation status. Data source: 2010, 2016 and 2021 French Perinatal National Surveys (ENP), and BFHI France……………………………………………………………………………………………………......………….**3**

**Supplementary Table S4.** Predicted exclusive (only breastmilk), mixed (breastmilk and formula) and any (addition of exclusive and mixed) breastfeeding rates in maternity units for mothers that delivered in a Baby-Friendly Hospital Initiative (BFHI) accredited maternity units (i.e. marginal predictions) for the per model. Data source: 2010, 2016 and 2021 French Perinatal National Surveys (ENP).………………………………………………………………………………………….…**4**

**Supplementary Table S5.** Predicted exclusive (only breastmilk), mixed (breastmilk and formula) and any (addition of exclusive and mixed)breastfeeding rates in maternity units for mothers that delivered in a non- Baby-Friendly Hospital Initiative (BFHI) accredited maternity units (i.e. marginal predictions) for the per model. Data source: 2010, 2016 and 2021 French Perinatal National Surveys (ENP)..………………………...............................................................................…**4**

**Supplementary Table S6.** Variables included and selected for each modelling step..………………………………...…….**5**

**Supplementary Table S7.** Difference in predicted exclusive (only breastmilk), mixed (breastmilk and formula) and any (addition of exclusive and mixed) breastfeeding rates in maternity units between each variable level and the reference level (i.e. marginal effect) of the variables in the final model, model five. For the continuous variables, we selected the mean as the reference value and selected specific values to calculate the marginal effect. Data source: 2010, 2016 and 2021 French Perinatal National Surveys (ENP)……………….……………………..**6-8**

**Supplementary Table S8**. Difference in predicted exclusive (only breastmilk), mixed (breastmilk and formula) and any (addition of exclusive and mixed) breastfeeding rates in maternity units between the Baby-Friendly Hospital Initiative (BFHI) accredited and non-accredited maternity units (i.e. marginal effect of the BFHI) for the variables in interaction with the BFHI in the final model, model five. Data source: 2010, 2016 and 2021 French Perinatal National Surveys (ENP).…………………………………………………………………………………….**9**

**Supplementary Table S9**. Number of maternity units that were Baby-Friendly Hospital Initiative (BFHI) accredited, in process of BFHI-accreditation, and not BFHI-accredited in 2010, 2016 and 2021……………………………………….…**10**

**Supplementary Table S10.** Adjusted predicted breastfeeding rates in maternity units that were Baby-Friendly Hospital Initiative (BFHI) accredited, in process of BFHI-accreditation, and non-BFHI maternity units in 2010, 2016 and 2021.....**10**

S**upplementary Figures…………………………………………………………………………………11-13**

**Supplementary Figure S1.** Selection process of the study population. Data for metropolitan France from the 2010, 2016 and 2021 French National Perinatal Surveys (ENP).………………………………………………………………….……..**11**

**Supplementary Figure S2.** Maps of predicted exclusive (only breastmilk) and mixed (breastmilk and formula) breastfeeding rates in maternity units by French administrative department. Data source: 2010, 2016 and 2021 French Perinatal National Surveys (ENP).……….…………………………………………………………………………….…….**12**

**Supplementary Figure S3.** Predicted mixed, breastmilk and formula, (grey triangle) and exclusive, only breastmilk, (black circle) breastfeeding rates in maternity units by French administrative department. Data source: 2010, 2016 and 2021 French Perinatal National Surveys (ENP).……….…………………………………………………………………….……………………………..…..……….**13**

**Suppplementary methods………………………………………………………………………………….14**

**Supplementary Table S1.**  The 12 recommendations of the Baby-Friendly Hospital Initiative (BFHI) in France, translated to English and in the original French version.

| **The 12 Recommendations** | *Les 12 Recommendations* |
| --- | --- |
| 1. Implement a policy for welcoming and supporting newborns and their families, formulated in writing and systematically communicated to all nursing staff. 2. Equip all nursing staff with the skills needed to implement this policy. 3. Inform all pregnant women about the advantages of breastfeeding and the practice of breastfeeding, whether or not they are registered with the hospital. Inform hospitalized pregnant women at risk of premature delivery or of having a sick child about the benefits of breastfeeding and how to manage lactation and breastfeeding. 4. Place the newborn in skin-to-skin contact with his or her mother immediately after birth for at least one hour, and encourage the mother to recognize when her newborn is ready to suckle, offering help if needed. For newborns born before 37 weeks' gestation, the aim is to maintain the greatest possible proximity between mother and newborn, whenever their medical condition permits. 5. Show breastfeeding mothers how to breastfeed and how to initiate and maintain lactation, even if they are separated from their newborn or if the newborn cannot suckle. Provide non-breastfeeding mothers with appropriate information on feeding their newborns. 6. Favor exclusive breast-feeding by not giving breast-fed newborns any food or drink other than breast milk, unless medically indicated.   * Give preference to the mother's milk, given untreated whenever possible, and give preference to milk from a lactarium if a supplement is required.   1. Leave the newborn with its mother 24 hours a day. Keep mother and newborn close to each other, giving priority to skin-to-skin contact and treating it as a form of care. 2. Encourage feeding on demand.   * Observe the behavior of premature and/or sick newborns to determine their ability to suckle. Suggest strategies for progressing towards independent feeding. For breastfed newborns, reserve the use of bottles and pacifiers for special situations.   1. For breastfed newborns, reserve the use of bottles and pacifiers for special situations. 2. Identify breastfeeding and other appropriate support associations and refer mothers to them as soon as they leave the hospital. Work in a network. 3. Protect families from commercial pressures by complying with the International Code of Marketing of Breast-milk Substitutes. 4. Follow practices that foster mother-child bonding during labor and delivery.   Criteria for premature and/or sick newborns apply to maternity and neonatal units. The icon * refers to neonatology only. | 1. *Adopter une politique d'accueil et d’accompagnement des nouveau-nés et de leur famille, formulée par écrit et systématiquement portée à la connaissance de tous les personnels soignants.* 2. *Donner à tous les personnels soignants les compétences nécessaires pour mettre en œuvre cette politique.* 3. *Informer toutes les femmes enceintes des avantages de l'allaitement au sein et de sa pratique, qu'elles soient suivies ou non dans l'établissement. Informer les femmes enceintes hospitalisées à risque d’accouchement prématuré ou de naissance d’un enfant malade des bénéfices de l’allaitement et de la conduite de la lactation et de l’allaitement.* 4. *Placer le nouveau-né en peau à peau avec sa mère immédiatement à la naissance pendant au moins une heure et encourager la mère à reconnaître quand son bébé est prêt à téter, en proposant de l’aide si besoin. Pour le nouveau-né né avant 37 SA, il s’agit de maintenir une proximité maximale entre la mère et le nouveauné, quand leur état médical le permet.* 5. *Indiquer aux mères qui allaitent comment pratiquer l'allaitement au sein et comment mettre en route et entretenir la lactation, même si elles se trouvent séparées de leur nouveau-né ou s’il ne peut pas téter. Donner aux mères qui n’allaitent pas des informations adaptées sur l’alimentation de leur nouveau-né* 6. *Privilégier l’allaitement maternel exclusif en ne donnant aux nouveau-nés allaités aucun aliment ni aucune boisson autre que le lait maternel, sauf indication médicale.*   ** Privilégier le lait de la mère, donné cru chaque fois que possible, et privilégier le lait de lactarium si un complément est nécessaire.*   1. *Laisser le nouveau-né avec sa mère 24 heures sur 24. Favoriser la proximité de la mère et du bébé, privilégier le contact peau à peau et le considérer comme un soin.* 2. *Encourager l'alimentation « à la demande » de l'enfant.*   ** Observer le comportement de l'enfant prématuré et/ou malade pour déterminer sa capacité à téter. Proposer des stratégies permettant de progresser vers l’alimentation autonome.*   1. *Pour les bébés allaités, réserver l'usage des biberons et des sucettes aux situations particulières.* 2. *Identifier les associations de soutien à l'allaitement maternel et autres soutiens adaptés et leur adresser les mères dès leur sortie de l'établissement. Travailler en réseau.* 3. *Protéger les familles des pressions commerciales en respectant le Code international de commercialisation des substituts du lait maternel.* 4. *Pendant le travail et l’accouchement, adopter des pratiques susceptibles de favoriser le lien mère-enfant*   *Les critères relatifs aux nouveau-nés prématurés et/ou malades sont applicables aux services de maternité et de néonatalogie. L’icône * ne concerne que la néonatalogie.* |

***Supplementary Table S2.*** *Survey questions of the French Perinatal National Surveys (ENP) used to measure breastfeeding at the maternity unit translated to English and in the original French version*

| Year | Survey question | *Question enquête* |
| --- | --- | --- |
| 2010 | How is your child fed today?   1. only breastmilk 2. only formula 3. mixed breastfeeding 4. unknown because infant was transferred*^a^* | *Aujourd’hui comment votre enfant est-il nourri ?*   1. *au lait maternel uniquement* 2. *au lait premier âge (ou LP) uniquement* 3. *allaitement mixte (lait maternel et lait premier âge ou LP)* 4. *inconnu car enfant transféré ^a^* |
| 2016 | How is your child fed today?   1. only breastmilk 2. only formula 3. mixed breastfeeding | *Aujourd’hui comment votre enfant est-il nourri ?*   1. *au lait maternel uniquement* 2. *au lait premier âge (ou lait préma) uniquement* 3. *allaitement mixte (lait maternel et lait premier âge ou lait préma)* |
| 2021 | How is your child fed today?   1. only breastmilk 2. only formula 3. mixed breastfeeding 4. unknown*^b^* | *Aujourd’hui comment votre enfant est-il nourri ?*   1. *au lait maternel (ou du lactarium) uniquement* 2. *au lait premier âge du commerce uniquement* 3. *allaitement mixte (lait maternel et lait premier âge du commerce)* 4. *inconnu ^b^* |

*a only in the ENP 2010.*

*b only in the ENP 2021. We considered this answer as missing and was later imputed.*

***Supplementary Table S3****. Distribution of the characteristics of maternity units by year and their Baby-Friendly Hospital Initiative (BFHI) accreditation status. Data source: 2010, 2016 and 2021 French Perinatal National Surveys (ENP), and BFHI France.*

| **Survey Year** | **2010** | | | **2016** | | | **2021** | | |
| --- | --- | --- | --- | --- | --- | --- | --- | --- | --- |
| **BFHI accreditation status** | **Non-accredited** | **Accredited** | ***P*^a^** | **Non-accredited** | **Accredited** | ***P*^a^** | **Non-accredited** | **Accredited** | ***P*^a^** |
|  | ***n=521*** | ***n=11*** |  | ***n=464*** | ***n=29*** |  | ***n=408*** | ***n=48*** |  |
| Size (annual deliveries): |  |  | 0.94 |  |  | 0.59 |  |  | 0.94 |
| <1000 | 199 (38.2%) | 4 (36.4%) |  | 190 (40.9%) | 15 (51.7%) |  | 173 (42.9%) | 23 (43.4%) |  |
| 1000-1999 | 188 (36.1%) | 5 (45.5%) |  | 156 (33.6%) | 7 (24.1%) |  | 122 (30.3%) | 19 (35.8%) |  |
| 2000-2999 | 94 (18.0%) | 2 (18.2%) |  | 66 (14.2%) | 3 (10.3%) |  | 54 (13.4%) | 5 (9.43%) |  |
| >=3000 | 40 (7.68%) | 0 (0.00%) |  | 52 (11.2%) | 4 (13.8%) |  | 54 (13.4%) | 6 (11.3%) |  |
| Status: |  |  | 0.04 |  |  | 0.01 |  |  | 0.12 |
| Public regional/univ | 49 (9.40%) | 0 (0.00%) |  | 46 (9.91%) | 1 (3.45%) |  | 46 (11.4%) | 1 (1.89%) |  |
| Other public | 287 (55.1%) | 9 (81.8%) |  | 267 (57.5%) | 20 (69.0%) |  | 237 (58.8%) | 35 (66.0%) |  |
| Non-profit private | 35 (6.72%) | 2 (18.2%) |  | 23 (4.96%) | 5 (17.2%) |  | 23 (5.71%) | 6 (11.3%) |  |
| Other private | 150 (28.8%) | 0 (0.00%) |  | 128 (27.6%) | 3 (10.3%) |  | 97 (24.1%) | 11 (20.8%) |  |
| Level of care: |  |  | 0.57 |  |  | 0.21 |  |  | 0.18 |
| I | 250 (48.0%) | 7 (63.6%) |  | 194 (41.8%) | 17 (58.6%) |  | 148 (36.7%) | 26 (49.1%) |  |
| II | 211 (40.5%) | 4 (36.4%) |  | 213 (45.9%) | 9 (31.0%) |  | 199 (49.4%) | 23 (43.4%) |  |
| III | 60 (11.5%) | 0 (0.00%) |  | 57 (12.3%) | 3 (10.3%) |  | 56 (13.9%) | 4 (7.55%) |  |

a The presented P values were obtained with Chi-squared tests.

***Supplementary Table S4.*** *Predicted exclusive (only breastmilk), mixed (breastmilk and formula) and any (addition of exclusive and mixed) breastfeeding rates in maternity units for mothers that delivered in a Baby-Friendly Hospital Initiative (BFHI) accredited maternity units (i.e. marginal predictions) for the per model. Data source: 2010, 2016 and 2021 French Perinatal National Surveys (ENP).*

|  | Breastfeeding | | |
| --- | --- | --- | --- |
| Model | **Exclusive** | **Mixed** | **Any** |
| 1 | 63.8 (61.7,66.0) | 5.2 (4.4,6.2) | 69.0 (66.9,71.2) |
| 2 | 64.5 (62.5,66.6) | 6.3 (5.3,7.5) | 70.8 (69.0,72.6) |
| 3 | 64.0 (62.1,66.0) | 6.4 (5.3,7.6) | 70.3 (68.4,72.2) |
| 4 | 63.7 (61.4,65.9) | 9.6 (8.0,11.3) | 73.3 (71.5,75.2) |
| 5 | 63.2 (61.0,65.4) | 9.6 (8.0,11.4) | 72.9 (71.0,74.8) |

**Supplementary Table S5.** Predicted exclusive (only breastmilk), mixed (breastmilk and formula) and any (addition of exclusive and mixed) breastfeeding rates in maternity units for mothers that delivered in a non- Baby-Friendly Hospital Initiative (BFHI) accredited maternity units (i.e. marginal predictions) for the per model. Data source: 2010, 2016 and 2021 French Perinatal National Surveys (ENP).

|  | Breastfeeding | | |
| --- | --- | --- | --- |
| Model | **Exclusive** | **Mixed** | **Any** |
| 1 | 57.6 (57.1,58.2) | 11.4 (11.1,11.8) | 69.0 (68.5,69.5) |
| 2 | 57.3 (56.5,58.0) | 11.6 (11.0,12.3) | 68.9 (68.3,69.4) |
| 3 | 57.7 (56.9,58.4) | 11.4 (10.9,12.1) | 69.1 (68.5,69.6) |
| 4 | 57.5 (56.6,58.1) | 11.3 (10.8,12.1) | 68.8 (68.2,69.3) |
| 5 | 57.5 (56.5,58.1) | 11.3 (10.8,12.1) | 68.8 (68.2,69.3) |

**Supplementary Table S6**. Variables included and selected for each modelling step.

| **Model 1** | **Model 2** | **Model 3** | **Model 4** | **Model 5** |
| --- | --- | --- | --- | --- |
|  | Model 1 + Maternal and newborn  characteristics | Model 2 + Maternity unit characteristics | Model 3 +French administrative department characteristics +  Spatial random effect | Model 4 + Interactions |
| **1.BFHI** | 1.Maternal age (linear) | **1.Size (linear)** | 1.Percentage of immigrants (linear) | BFHI x |
| **2.Survey year** | **2.Maternal level of education** | **2.Status** | 2.Percentage of university graduates | 1.Survey year |
|  | **3.Maternal country of birth** | 3.Level of care | 3.Percentage of urban population | 2.Maternal level of education |
|  | 4.Marital status |  |  | 3.Maternal country of birth |
|  | **5.Average monthly household income** |  |  | 4.Average monthly household income |
|  | 6.Employment during pregnancy |  |  | 5.Parity |
|  | **7.Parity** |  |  | 6.Mode of delivery |
|  | **8. Pre-pregnancy BMI** |  |  | 7.Time between giving birth and the survey interview |
|  | **9.Mode of delivery** |  |  | **8.Birthweight** |
|  | **10.Time between delivery and interview** |  |  | 9.Gestational age |
|  | **11.Birthweight** |  |  |  |
|  | **12.Gestational age** |  |  |  |
|  | **13.Neonatal transfer** |  |  |  |

**Only adjusted for in comparison of exclusive breastfeeding to mixed breastfeeding**

Only adjusted for in comparison of exclusive breastfeeding to no breastfeeding

**Adjusted for in both comparisons**

Not included in any of the comparisons

The final models included the following variables:

**Sub-model of exclusive breastfeeding to mixed breastfeeding:**

**BFHI, survey year, maternal level of education, maternal country of birth, average monthly household income, parity, pre-pregnancy BMI, mode of delivery, time between delivery and interview, birthweight, gestational age, neonatal transfer, size of maternity unit, status of maternity unit, BFHI x birthweight**

Sub-model of exclusive breastfeeding to no breastfeeding:

BFHI, survey year, maternal age (linear), maternal level of education, maternal country of birth, marital status, parity, pre-pregnancy BMI, mode of delivery, time between delivery and interview, birthweight, gestational age, neonatal transfer, size of maternity unit (linear), status of maternity unit, percentage of immigrants (linear), percentage of university graduates, BFHI x maternal level of education

***Supplementary Table S7.*** *Difference in predicted exclusive (only breastmilk), mixed (breastmilk and formula) and any (addition of exclusive and mixed) breastfeeding rates in maternity units between each variable level and the reference level (i.e. marginal effect) of the variables in the final model, model five. For the continuous variables, we selected the mean as the reference value and selected specific values to calculate the marginal effect. Data source: 2010, 2016 and 2021 French Perinatal National Surveys (ENP).*

|  |  |  | | **Breastfeeding** | | |
| --- | --- | --- | --- | --- | --- | --- |
| **Variable** | **Value** | **Reference** | **Exclusive** | | **Mixed** | **Any** |
| Year | 2016 | 2010 | -9.7 (-11.0,-8.3) | | 5.6 (4.8,6.4) | -4.1 (-5.3,-2.9) |
|  | 2021 |  | -9.4 (-10.7,-8.2) | | 6.1 (5.4,6.9) | -3.3 (-4.5,-2.1) |
| Maternal age | 18 | 30 | -2.8 (-4.0,-1.8) | | -0.5 (-0.7,-0.3) | -3.3 (-4.7,-2.1) |
|  | 20 |  | -2.4 (-3.3,-1.5) | | -0.4 (-0.5,-0.2) | -2.7 (-3.9,-1.7) |
|  | 25 |  | -1.2 (-1.6,-0.7) | | -0.2 (-0.3,-0.1) | -1.4 (-1.9,-0.9) |
|  | 35 |  | 1.1 (0.7,1.6) | | 0.2 (0.1,0.3) | 1.3 (0.9,1.9) |
|  | 40 |  | 2.3 (1.5,3.1) | | 0.4 (0.2,0.5) | 2.6 (1.7,3.7) |
|  | 45 |  | 3.3 (2.2,4.6) | | 0.5 (0.4,0.8) | 3.9 (2.5,5.4) |
| Country of birth | African country | France | 13.8 (12.1,15.3) | | 12.1 (10.8,13.4) | 25.9 (24.6,27.1) |
|  | Other country |  | 15.0 (13.1,16.8) | | 5.7 (4.4,7.0) | 20.6 (19.0,22.3) |
| Marital status | Married | Not married | 5.2 (4.4,6.1) | | 0.9 (0.7,1.0) | 6.1 (5.1,7.1) |
| Average monthly household income (€) | 1500-2999 | <1500 | 2.0 (1.3,2.7) | | -2.8 (-3.9,-1.8) | -0.9 (-1.2,-0.6) |
|  | >=3000 |  | 3.6 (2.7,4.4) | | -5.1 (-6.3,-3.9) | -1.5 (-1.9,-1.2) |
| Parity (number of births, including this delivery) | Primiparous | 0 | -5.8 (-7.0,-4.6) | | -1.4 (-2.2,-0.7) | -7.2 (-8.3,-6.1) |
|  | 2 |  | -4.4 (-5.9,-2.9) | | -1.3 (-2.3,-0.4) | -5.8 (-7.3,-4.3) |
|  | 3 |  | -3.0 (-5.6,-0.5) | | -0.0 (-1.5,1.4) | -3.0 (-5.5,-0.7) |
|  | 4 or more |  | -8.6 (-12.1,-5.4) | | 4.3 (2.3,6.5) | -4.3 (-7.8,-1.2) |
| Pre-pregnancy BMI (kg/m²) | 15 | 24 | 0.6 (-3.1,4.5) | | -2.7 (-3.9,-1.4) | -2.1 (-6.5,2.3) |
|  | 20 |  | 1.2 (-0.0,2.4) | | -1.3 (-2.1,-0.5) | -0.2 (-1.8,1.6) |
|  | 25 |  | -0.5 (-0.7,-0.2) | | 0.4 (0.2,0.5) | -0.1 (-0.3,0.1) |
|  | 30 |  | -3.4 (-4.7,-1.8) | | 2.3 (1.2,3.3) | -1.1 (-3.0,0.8) |
|  | 35 |  | -6.5 (-9.6,-3.6) | | 3.5 (0.4,6.8) | -3.0 (-8.7,2.5) |
|  | 40 |  | -9.0 (-14.4,-4.3) | | 3.9 (-1.3,10.1) | -5.0 (-15.1,4.6) |
| Time between delivery and interview (days) | 0 | 1 | 1.2 (-6.7,6.7) | | -4.1 (-7.5,0.3) | -2.9 (-14.3,6.6) |
|  | 2 |  | -3.6 (-5.7,-1.9) | | 4.8 (1.8,8.4) | 1.2 (-4.0,6.2) |
|  | 4 |  | -10.3 (-20.6,-3.2) | | 0.1 (-6.3,8.9) | -10.2 (-26.9,5.0) |
|  | 6 |  | -22.3 (-52.8,2.8) | | -4.0 (-10.7,17.2) | -26.3 (-63.3,17.4) |
|  | 8 |  | -29.9 (-58.8,10.5) | | -5.8 (-10.9,23.3) | -35.6 (-69.6,24.4) |
|  | 10 |  | -33.2 (-59.4,16.3) | | -6.6 (-10.9,24.5) | -39.9 (-70.2,27.1) |
| Mode of delivery | Instrumental | Spontaneous vaginal | -4.3 (-6.0,-2.7) | | 0.7 (-0.3,1.7) | -3.7 (-5.2,-2.2) |
|  | Caesarean Section |  | -5.6 (-6.9,-4.2) | | 3.4 (2.6,4.3) | -2.2 (-3.5,-1.0) |
| Gestational age (weeks) | 37 | 39 | -1.4 (-3.1,0.2) | | 0.9 (-0.2,2.0) | -0.5 (-2.4,1.2) |
|  | 38 |  | -1.0 (-2.0,-0.1) | | 0.3 (-0.2,0.9) | -0.7 (-1.8,0.4) |
|  | 40 |  | 1.5 (0.4,2.6) | | 0.3 (-0.0,0.6) | 1.7 (0.4,3.1) |
|  | 41 |  | 2.4 (0.8,4.0) | | 0.7 (-0.1,1.7) | 3.2 (1.2,5.2) |
|  | 42 |  | 2.9 (0.5,5.2) | | 1.4 (0.0,2.8) | 4.3 (1.8,6.8) |
| Neonatal transfer | Yes | No | -11.0 (-13.6,-8.6) | | 8.1 (6.4,9.9) | -2.9 (-5.2,-0.6) |
| Baby-Friendly Hospital Initiative Accreditation | Yes | No | 5.9 (3.5,8.1) | | -1.7 (-3.3,0.1) | 4.2 (2.2,6.2) |
| Maternity unit size (annual deliveries) | 500 | 2133 | 0.2 (-1.0,1.5) | | -1.3 (-2.2,-0.3) | -1.1 (-2.3,0.1) |
|  | 1000 |  | 0.3 (-0.6,1.2) | | -0.7 (-1.4,-0.0) | -0.4 (-1.5,0.7) |
|  | 2000 |  | 0.1 (-0.1,0.2) | | -0.0 (-0.1,0.1) | 0.0 (-0.2,0.3) |
|  | 3000 |  | -0.3 (-1.3,0.8) | | -0.2 (-0.7,0.3) | -0.5 (-2.0,0.9) |
|  | 4000 |  | 0.0 (-2.0,1.9) | | -0.8 (-1.9,0.4) | -0.7 (-3.4,2.0) |
|  | 5000 |  | 1.1 (-1.3,3.3) | | -1.4 (-3.0,0.4) | -0.3 (-3.6,2.9) |
|  | 6000 |  | 2.2 (-0.7,4.9) | | -2.2 (-4.4,0.5) | -0.0 (-3.9,3.9) |
| Maternity unit status | Other public | Public regional/univ | 1.5 (-0.3,3.3) | | -3.0 (-4.2,-1.9) | -1.5 (-3.2,0.2) |
|  | Non-profit private |  | 3.9 (1.5,6.1) | | -2.9 (-4.4,-1.3) | 1.1 (-1.1,3.3) |
|  | Other private |  | 0.7 (-1.3,2.4) | | -2.8 (-4.0,-1.7) | -2.2 (-3.9,-0.5) |
| Immigrants in a French administrative department (%) | 5 | 10 | -1.3 (-2.5,-0.2) | | -0.2 (-0.4,-0.0) | -1.5 (-2.9,-0.2) |
|  | 15 |  | 1.2 (0.2,2.4) | | 0.2 (0.0,0.4) | 1.4 (0.2,2.8) |
|  | 20 |  | 2.4 (0.3,4.6) | | 0.4 (0.1,0.8) | 2.8 (0.4,5.4) |
|  | 25 |  | 3.5 (0.5,6.8) | | 0.6 (0.1,1.1) | 4.1 (0.6,7.9) |
|  | 30 |  | 4.7 (0.7,8.7) | | 0.8 (0.1,1.4) | 5.4 (0.8,10.2) |
| University graduates in a French administrative department (%) | 10 | 29 | -4.3 (-8.0,-1.0) | | -0.7 (-1.3,-0.2) | -5.0 (-9.2,-1.2) |
|  | 20 |  | -1.9 (-3.3,-0.5) | | -0.3 (-0.5,-0.1) | -2.3 (-3.9,-0.6) |
|  | 30 |  | 0.1 (-0.0,0.3) | | 0.0 (-0.0,0.1) | 0.2 (-0.0,0.4) |
|  | 40 |  | 0.3 (-1.3,2.1) | | 0.1 (-0.2,0.3) | 0.4 (-1.5,2.4) |
|  | 50 |  | -1.2 (-4.0,1.8) | | -0.2 (-0.6,0.3) | -1.4 (-4.6,2.1) |
|  | 60 |  | -3.5 (-7.8,0.9) | | -0.6 (-1.3,0.1) | -4.0 (-9.1,1.0) |

***Supplementary Table S8****. Difference in predicted exclusive (only breastmilk), mixed (breastmilk and formula) and any (addition of exclusive and mixed) breastfeeding rates in maternity units between the Baby-Friendly Hospital Initiative (BFHI) accredited and non-accredited maternity units (i.e. marginal effect of the BFHI) for the variables in interaction with the BFHI in the final model, model five. Data source: 2010, 2016 and 2021 French Perinatal National Surveys (ENP).*

|  |  | Breastfeeding | | |
| --- | --- | --- | --- | --- |
| Variable | **Value** | **Exclusive** | **Mixed** | **Any** |
| Birthweight (g) | 2000 | 19.2 (10.0,27.5) | -12.9 (-25.4,2.0) | 6.3 (-3.1,15.7) |
|  | 2500 | 14.9 (10.0,19.9) | -5.7 (-12.5,1.9) | 9.2 (1.9,15.9) |
|  | 3000 | 9.5 (6.0,12.7) | -1.0 (-3.0,1.3) | 8.5 (4.3,12.5) |
|  | 3500 | 3.7 (1.4,6.0) | -0.6 (-2.3,1.2) | 3.1 (0.6,5.5) |
|  | 4000 | -0.5 (-7.0,5.4) | -3.5 (-6.3,-0.6) | -4.0 (-12.1,3.9) |
| Level of education | No/primary | -6.2 (-18.6,6.7) | -3.8 (-6.4,-1.0) | -10.0 (-24.7,5.1) |
|  | Lower secondary | 6.1 (2.6,9.8) | -1.4 (-2.8,0.2) | 4.7 (1.0,8.4) |
|  | Upper secondary | 6.1 (2.5,9.6) | -1.6 (-3.0,0.1) | 4.5 (0.8,8.2) |
|  | 1-2 years univ | 2.5 (-1.1,5.9) | -2.0 (-3.4,-0.6) | 0.5 (-3.3,3.9) |
|  | >2 years univ | 7.8 (5.2,10.1) | -1.5 (-2.9,-0.1) | 6.3 (3.9,8.5) |

**Supplementary Table S9.** Number of maternity units that were Baby-Friendly Hospital Initiative (BFHI) accredited, in process of BFHI-accreditation, and not BFHI-accredited in 2010, 2016 and 2021

|  | 2010 | 2016 | 2021 | Total |
| --- | --- | --- | --- | --- |
| Accredited | 11 (2.07%) | 29 (5.88%) | 48 (10.53%) | 88 |
| In process of  BFHI-accreditation | 20 (3.76%) | 40 (8.11%) | 31 (6.80%) | 91 |
| Non-accredited | 501 (94.17%) | 424 (86.00%) | 377 (82.68%) | 1 302 |
| Total | 532 | 493 | 456 |  |

***Supplementary Table S10****. Adjusted predicted breastfeeding rates in maternity units that were BFHI accredited, in process of BFHI-accreditation, and non-BFHI maternity units in 2010, 2016 and 2021*

|  | Accredited | In process of  BFHI-accreditation | Non-accredited |
| --- | --- | --- | --- |
| Exclusive breastfeeding | 63.6 (61.1,66.0) | 60.9 (58.7,63.0) | 57.2 (56.3,58.0) |
| Mixed breastfeeding | 9.4 (7.9,11.1) | 8.5 (7.2,10.0) | 11.5 (10.9,12.4) |
| Any breastfeeding | 73.0 (71.1, 74.7) | 69.4 (67.7,71.3) | 68.7 (68.1,69.2) |

**Supplementary Figure S1.** Selection process of the study population. Data for metropolitan France from the 2010, 2016 and 2021 French National Perinatal Surveys (ENP).


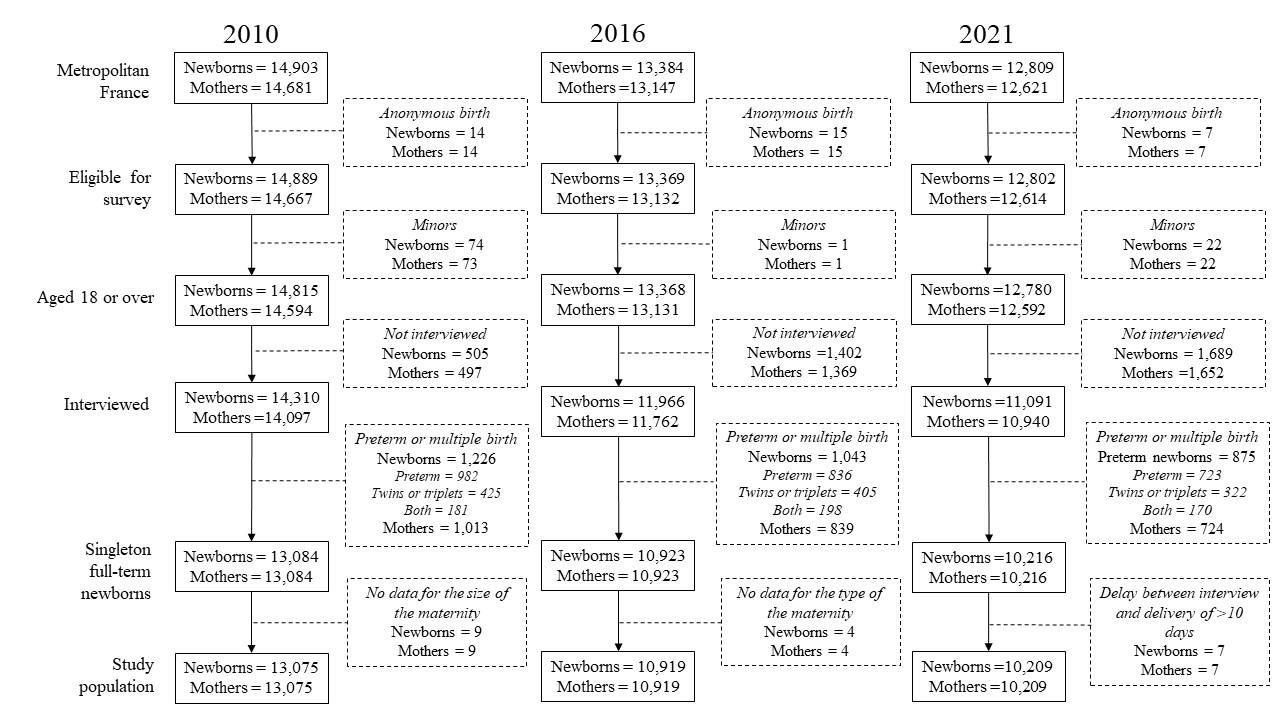


**Supplementary Figure S2.** Maps of predicted exclusive (only breastmilk) and mixed (breastmilk and formula) breastfeeding rates in maternity units by French administrative department. Data source: 2010, 2016 and 2021 French Perinatal National Surveys (ENP).


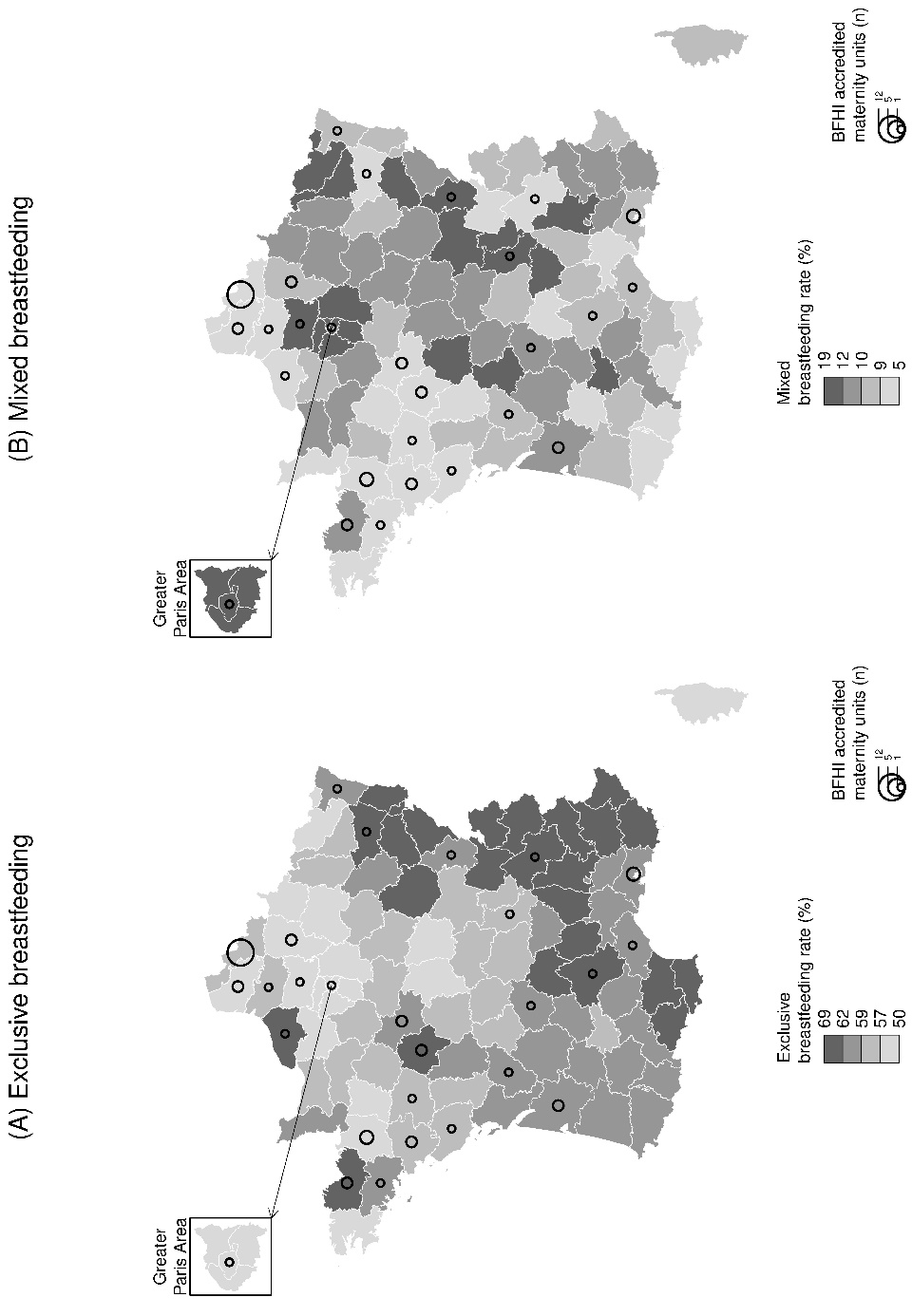


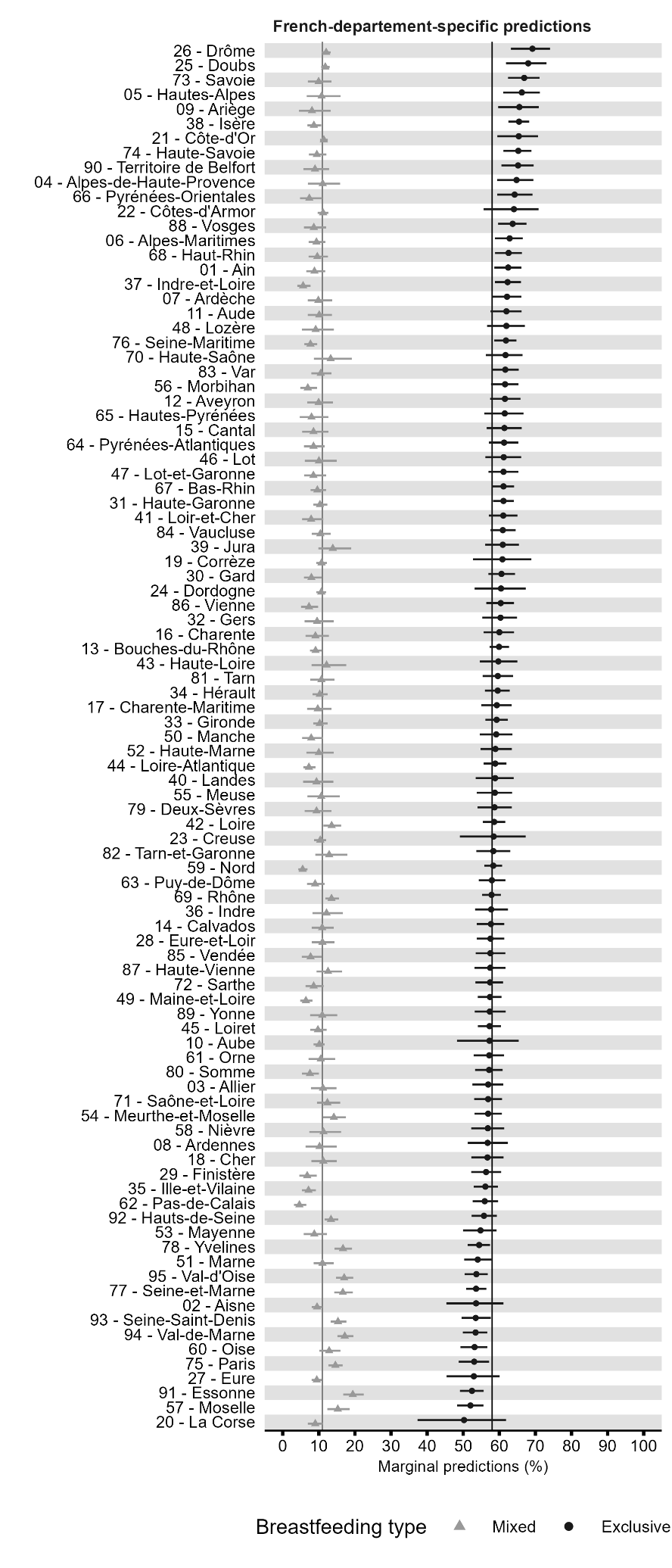
**Supplementary Figure S3.** Predicted mixed, breastmilk and formula, (grey triangle) and exclusive, only breastmilk, (black circle) breastfeeding rates in maternity units by French administrative department. Data source: 2010, 2016 and 2021 French Perinatal National Surveys (ENP)

**French-administrative-deparment specific predictions**

***Supplementary methods***

*Explanatory variables*

- Individual: maternal age, maternal level of education (no/primary, lower secondary, upper secondary, 1-2 years of university, >2 years of university), maternal country of birth (France, African country, Other country), marital status (not married, married), average monthly household income (<1500€, 1500-2999€, >=3000€), employment during pregnancy (no, yes), parity (i.e., number of births before this delivery), pre-pregnancy body mass index (BMI in kg/m^2^), mode of delivery (spontaneous vaginal, instrumental, caesarean section), the time between giving birth and the survey interview (in days), birthweight (g), gestational age (weeks), and neonatal transfer (no, yes).
- Maternity unit: size (annual number of deliveries), status (public regional or university, other public, non-profit private, other private), level of care (I: care for low-risk newborns, no neonatal unit; II: intermediate special care units; and III: neonatal intensive care units). In 2021, the participating 6 birthing centres were given the status of the maternity unit they were attached to.
- French administrative department of the maternity unit (metropolitan France is divided into 13 admnisitrative regions and 96 administrative departments (21)): the percentage of immigrants in 2010, 2016 and 2021 in the French administrative department (22-24), the percentage of residents aged 16 years-old and over with a graduate or post-graduate education in 2010, 2016 and 2019 (25-27), and the percentage of urban population based on the classification of rural and urban areas in 2010 and 2020 (28, 29) and the population of 2010, 2016 and 2020 (30-32).

*Imputation with the missForest method*

The variables included in the missForest model were: breastfeeding status, maternal age, maternal level of education, maternal country of birth, marital status, average monthly household income, employment during pregnancy, parity, pre-pregnancy body mass index, mode of delivery, the time between giving birth and the survey interview, birthweight (g), gestational age (weeks), and neonatal transfer, maternity unit size, status, level of care, and BFHI accreditation status, region, the percentage of immigrants, the percentage of residents aged 16 years-old and over with a graduate or post-graduate education, and the percentage of urban population. Of note, the French administrative department variable, containing too many (96) modalities, was replaced by the “region” variable, with 13 modalities.
